# Assessment of idiopathic inflammatory myopathy using a deep learning method for muscle T2 mapping segmentation

**DOI:** 10.1007/s00330-022-09254-9

**Published:** 2022-11-18

**Authors:** Fengdan Wang, Shuang Zhou, Bo Hou, Francesco Santini, Ling Yuan, Ye Guo, Jinxia Zhu, Tom Hilbert, Tobias Kober, Yan Zhang, Qian Wang, Yan Zhao, Zhengyu Jin

**Affiliations:** 1 Department of Radiology, State Key Laboratory of Complex Severe and Rare Diseases, Peking Union Medical College Hospital, Chinese Academy of Medical Sciences & Peking Union Medical College, Beijing, China; 2 Department of Rheumatology and Clinical Immunology, State Key Laboratory of Complex Severe and Rare Diseases, Peking Union Medical College Hospital, Chinese Academy of Medical Sciences & Peking Union Medical College, Beijing, China; 3 Department of Research & Analytic Services, University Hospital Basel, Petersgraben 4, CH-4031 Basel, Switzerland; 4 Radiological Physics, University Hospital Basel, Basel, Switzerland; 5 Department of Biomedical Engineering, University of Basel, Allschwil, Switzerland; 6 Department of Clinical Laboratory, State Key Laboratory of Complex Severe and Rare Diseases, Peking Union Medical College Hospital, Chinese Academy of Medical Sciences & Peking Union Medical College, Beijing, China; 7 MR Collaboration, Siemens Healthcare Ltd., Beijing, China; 8 Advanced Clinical Imaging Technology, Siemens Healthcare AG, Lausanne, Switzerland; 9 Department of Radiology, Lausanne University Hospital and University of Lausanne, Lausanne, Switzerland; 10 École Polytechnique Fédérale de Lausanne, Lausanne, Switzerland; 11 Department of Radiology, Peking Union Medical College Hospital, No. 1 Shuaifuyuan, Dongcheng District, Beijing, China

**Keywords:** Magnetic resonance imaging, Thigh muscles, Deep learning, Myositis, Idiopathic inflammatory myopathy

## Abstract

**Objective:**

To investigate the utility of an automatic deep learning (DL) method for segmentation of T2 maps in patients with idiopathic inflammatory myopathy (IIM) against healthy controls, and also the association of quantitative T2 values in patients with laboratory and pulmonary findings.

**Methods:**

Structural MRI and T2 mapping of bilateral thigh muscles from patients with IIM and healthy volunteers were segmented using dedicated software based on a pre-trained convolutional neural network. Incremental and federated learning were implemented for continuous adaptation and improvement. Muscle T2 values derived from DL segmentation were compared between patients and healthy controls, and T2 values of patients were further analyzed with serum muscle enzymes, and interstitial lung disease (ILD) which was diagnosed and graded based on chest HRCT.

**Results:**

Overall, 64 patients (27 patients with dermatomyositis, 29 with polymyositis, and 8 with antisynthetase syndrome (ASS)) and 10 healthy controls were included. By using DL-based muscle segmentation, T2 values generated from T2 maps accurately differentiated patients from those of controls (*p* < 0.001) with a cutoff value of 36.4 ms (sensitivity 96.9%, and specificity 100%). In patients with IIM, muscle T2 values positively correlated with all the serum muscle enzymes (all *p* < 0.05). ILD score of patients with ASS was markedly higher than that of those without ASS (*p* = 0.011), while dissociation between the severity of muscular involvement and ILD was observed (*p* = 0.080).

**Conclusion:**

Automatic DL could be used to segment thigh muscles and help quantitatively assess muscular inflammation of IIM through T2 mapping.

**Key Points:**

• *Muscle T2 mapping automatically segmented by deep learning can differentiate IIM from healthy controls.*

• *T2 value, an indicator of active muscle inflammation, positively correlates with serum muscle enzymes.*

*• T2 mapping can detect muscle disease in patients with normal muscle enzyme levels.*

**Supplementary Information:**

The online version contains supplementary material available at 10.1007/s00330-022-09254-9.

## Introduction

Idiopathic inflammatory myopathy (IIM) is a group of immune-mediated myopathies, of which dermatomyositis (DM) and polymyositis (PM) are the two leading types characterized by the shared features of symmetrical proximal muscle weakness and evidence of muscle inflammation [1]. IIM is highly debilitating and subject to relapse. It is associated with a variety of complications such as interstitial lung disease (ILD), cardiac arrhythmias, and malignancy [2, 3]. Although muscle weakness is the most common feature of IIM, this symptom usually develops in a subacute fashion, with gradual worsening over a period of several months before confirmed diagnosis.

To detect muscle inflammation, magnetic resonance imaging (MRI) has been incorporated into the workflow of diagnosing and monitoring IIM because of its non-invasiveness and high soft-tissue resolution [4, 5]. Increased signal intensity on inversion recovery or fat-suppressed T2-weighted image (WI) localizes muscle edema, and T1WI could detect fatty infiltration in muscles since fatty replacement shortens T1 time. Therefore, MRI is helpful to depict the involvement pattern of muscles, optimize the biopsy site, and be repeatedly performed to monitor the treatment response [4, 5].

Because the evaluation of signal changes in structural MR image is subjective and difficult to quantify, a quantitative parameter, T2 mapping, has been investigated to assess the active muscle inflammation in IIM. Increased thigh muscle T2 values were observed in juvenile and adult patients with active IIM compared with those with inactive disease or healthy volunteers [6, 7]. With the advent of rapid acquisition techniques, dedicated T2 mapping of bilateral thigh muscles can be acquired within 3 min at high resolution, which greatly increases the feasibility of applying T2 mapping clinically [8]. However, for post-processing, most previous studies measured muscle T2 values by manually drawing the regions of interest (ROIs) on T2 maps which is time-consuming. Moreover, such ROIs miss a large amount of data and are subject to sampling errors for the following reasons: up to 24 muscles are present in one slice, one patient can have dozens of slices, the area of a single ROI is usually less than 100 mm^2^, and the muscle involvement of IIM is heterogeneous in terms of both extent and signal intensity.

Deep learning (DL) can learn and improve algorithms automatically and can potentially be used to analyze all muscles in all slices [9]. Most importantly, DL can perform muscle segmentation automatically. Nevertheless, using muscle T2 mapping, segmented by DL in IIM, has not been described. This study sought to validate thigh muscle T2 values derived from automatic DL segmentation in patients with IIM against healthy controls. It also aimed to evaluate quantitative T2 values in patients with IIM, with regard to laboratory and pulmonary findings.

## Methods

### Study population

This retrospective study was approved by the Institutional Review Board, and written informed consent was waived. IIM patients were added to the study from November 2016 to July 2019. Inclusion criteria were in accordance with Bohan and Peter criteria [10, 11] and dedicated thigh MRIs were performed to evaluate active IIM. Antisynthetase syndrome (ASS) is defined by the presence of antibodies directed against one of several aminoacyl-transfer RNA (tRNA) synthetases, and the presence of myositis and multiple-organ involvement, primarily ILD [12]. Ten healthy participants who were free of systemic or musculoskeletal diseases were included as a control group.

### Laboratory and pulmonary assessments

Serum muscle enzymes were recorded from chart review, including creatine kinase (CK), alanine aminotransferase (ALT), aspartate aminotransferase (AST), and lactate dehydrogenase (LDH). Two radiologists evaluated chest high-resolution computed tomography (HRCT) images in a consensus manner to determine whether there was ILD and further graded the severity and extent of ILD as previously described [13]. By using a 5-point Likert score, the severity of ILD was graded into no ILD (0), minimal (1), mild (2), moderate (3), or severe (4) (Supplementary Figure 1); the extent of ILD was graded from 0: no opacity, score 1: 10 to 25%, score 2: 25 to 50%, score 3: 50 to 75%, and score 4: 75 to 100% of the lung was affected by ILD. The final ILD score was the sum of the two scores.

### MR imaging

Bilateral thigh muscles were scanned by a 3-T MR scanner (MAGNETOM Skyra, Siemens Healthcare, Erlangen, Germany) using an 18-channel body coil. The structural MRI included coronal T1WI (repetition time/echo time (TR/TE), 6.73/2.46 ms; slice thickness, 1 mm; and gap, 0 mm), coronal T2WI with Dixon (TR/TE, 3050/119 ms; slice thickness, 5 mm; and gap, 1 mm), and axial T2WI with Dixon (TR/TE, 5330/51 ms; slice thickness, 5 mm; and gap, 1 mm). After the non-gap acquisition of coronal T1WI, a set of 5-mm-thick axial T1WI was then reconstructed for clinical evaluation. After the acquisition of T2WI with Dixon, four sets of images, including in-phase, opposed-phase, fat-only, and water-only images, were automatically generated.

Dedicated axial T2 mapping was obtained with chemical-shift-selective fat saturation (TR, 6690 ms; TE, 10–100 ms, ΔTE = 10 ms; FOV, 420 × 231 mm^2^; base resolution, 254 × 84; slice thickness, 5 mm; and gap, 1 mm; undersampling factor, 6; acquisition time, 1 min 49 s) using a prototype sequence. T2 mapping was accelerated by a previously reported technique [14], GRAPPATINI, a novel combination of MARTINI (model-based accelerated relaxometry by iterative nonlinear inversion) and GRAPPA (generalized autocalibrating partial parallel acquisition), allowing for block-based Cartesian undersampling in *k*-space. A GRAPPA acceleration factor of 2 with an additional MARITINI undersampling factor of 5 was used, resulting in a 10-fold total acceleration. Therefore, the acquisition time for this T2 mapping sequence was only 2 min and 18 s.

### T2 measurement segmented by deep learning

An open-source software package termed “Deep Anatomical Federated Network” (DAFNE, https://dafne.network/) was used for the segmentation of anatomical images [9]. This software implemented a pre-trained customized Vnet deep learning network for automated segmentation of the thigh muscles and a user interface for checking and refining the automated segmentation. The software also performed incremental learning to continuously improve the performance of the segmentation on the local datasets and interfaced with a central server that federates the improved models provided by all the users of the software. As a result, DAFNE generates segmentation maps with Dice scores ranging from 0.89 to 0.95, with respect to “ground truth” manually segmented labelled images.

**Fig. 1:**
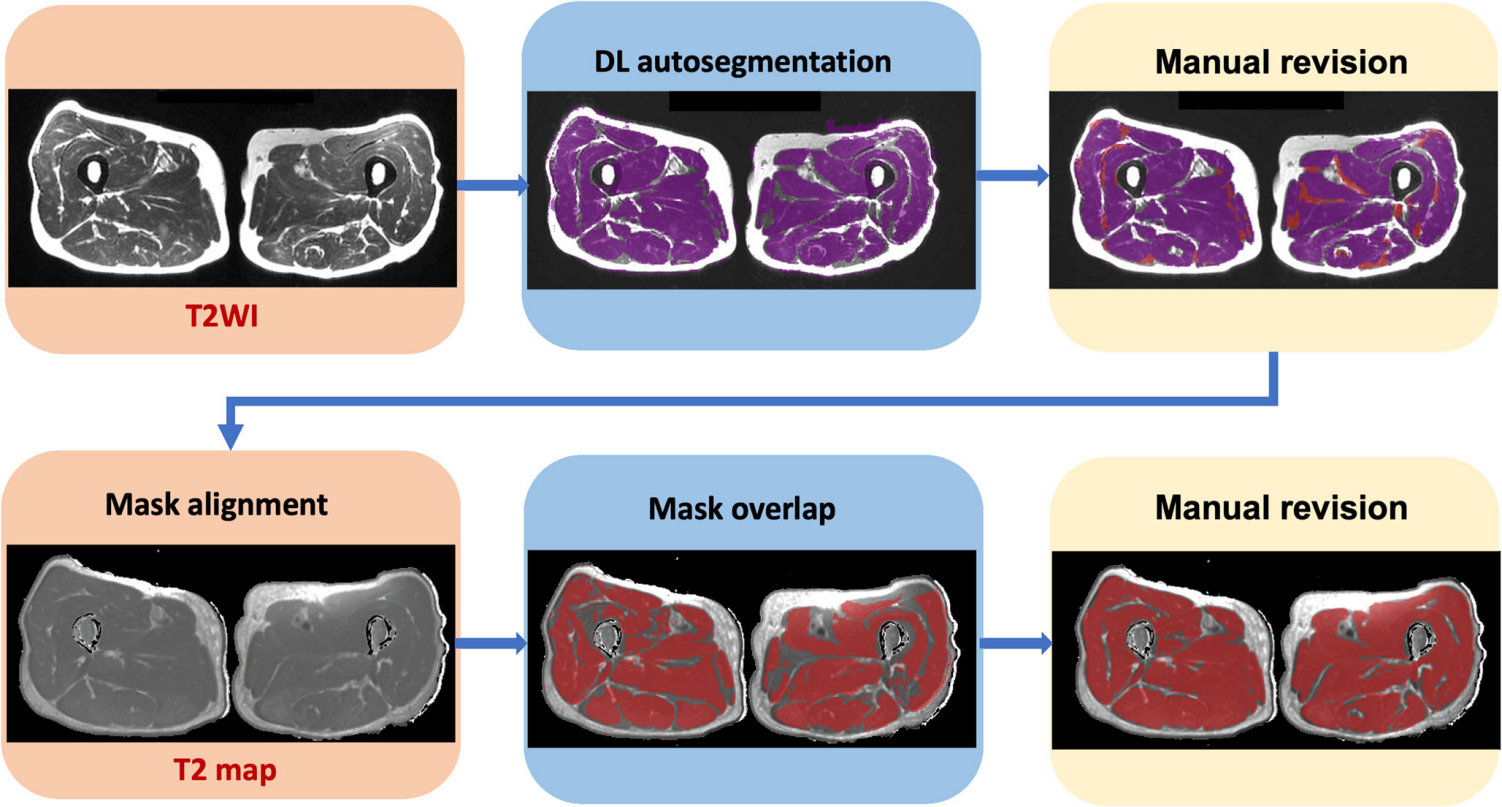
Demonstration of muscle T2 measurement segmented by deep learning (DL). The T2WI in-phase images were used as the structural images for auto-segmentation, with segmentation results marked in purple. Minor manual revision was then performed to erase unnecessary regions, and add missed muscle areas which were marked in red. This revised segmentation was saved as masks which were imported and matched to the corresponding slices of T2 map images by the alignment module. Subsequently, the final region of interest (marked in dark red) was achieved after manual revision if you want and muscle T2 value of the bilateral thigh muscles was provided

In our study, the segmentation of the muscle tissue from the fat and bone was performed on the T2WI Dixon images, and the produced ROIs were subsequently realigned to the T2 mapping images by the same software using the DICOM orientation information. Finally, a manual adaptation to account for misalignment between sequences was performed to obtain the final segmentation of the T2 maps. Average T2 values were extracted from the aligned ROIs and used for the subsequent analysis (Fig. 1).

### Statistical analyses

The efficiency of T2 mapping between patients and healthy controls was evaluated using receiver operating characteristic (ROC), and the cutoff of ROC analysis was also calculated. The Spearman correlation coefficient was used to assess the correlation between T2 values, and laboratory and pulmonary findings. The differences of T2 value and ILD score between the two groups were compared using the Mann-Whitney *U* test. A *p* < 0.05 was considered statistically significant. Data were analyzed using SPSS v.26.0 software (SPSS Inc.).

## Results

### Study population

A total of 74 subjects (64 patients and 10 healthy controls) were included in this study. Twenty-four patients underwent open muscle biopsy, one patient underwent skin biopsy, and another one received both muscle and skin biopsy. Finally, the 64 patients consisted of 27 patients with DM (12 definite, and 14 probable DM), 29 with PM (14 definite, and 15 probable PM), and 8 with ASS; there were 26 males and 38 females (male-to-female ratio, 1:1.5); the mean age was 44.3 years (range: 19–72 years); and the mean disease duration was 22.2 months (range: 1–144 months). Thirty-seven patients were untreated, and the other 27 patients relapsed during treatment with glucocorticoids and immunosuppressive drugs, which means all of the participants had active IIM. The healthy control group consisted of one male and nine females, and the mean age was 36.6 years (range: 25–63 years).

### Muscle T2 map segmented using deep learning–differentiated IIM from healthy controls

**Fig. 2:**
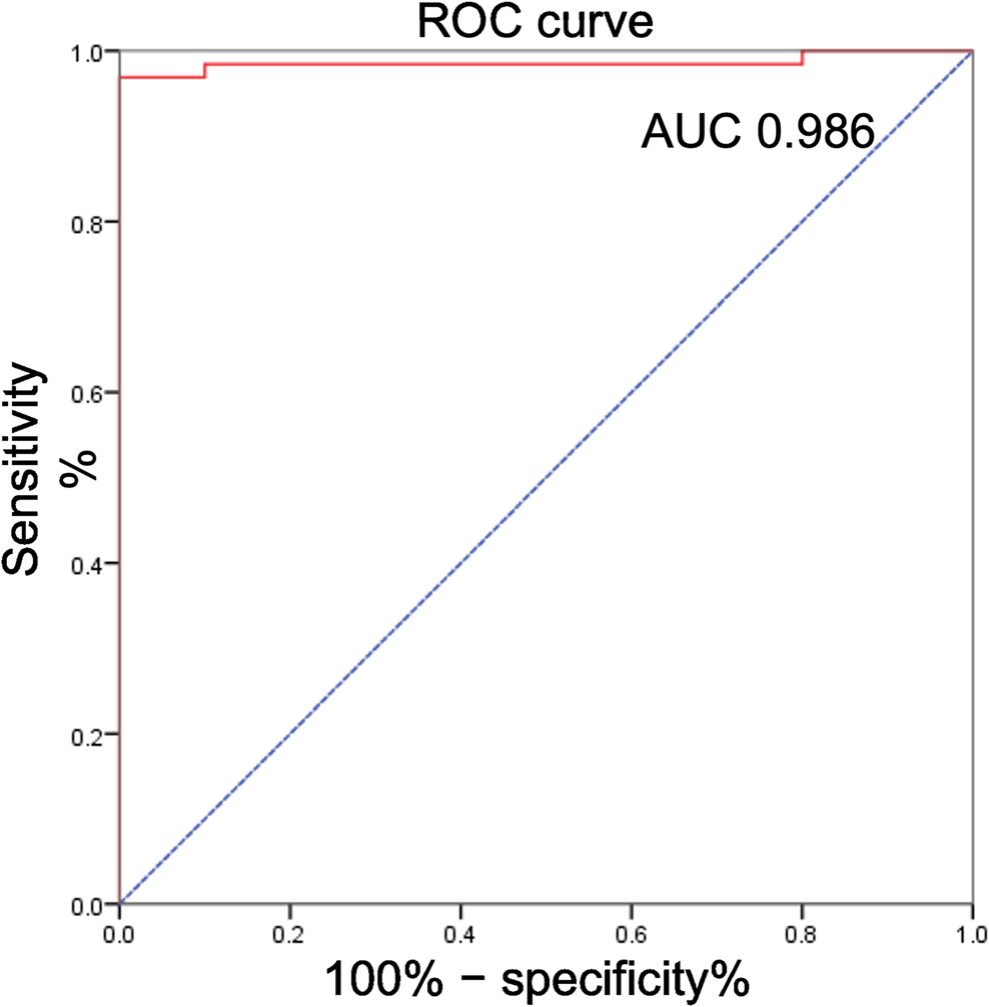
The ROC curve (red line) for the diagnostic performance of T2 mapping. The ROC curve (red line) for the diagnostic performance of T2 mapping in the differentiation of patients with idiopathic inflammatory myopathy from healthy controls. The area under the curve (AUC) was 0.986

To determine whether muscle T2 maps, segmented by DL, could detect muscle inflammation of IIM, thigh muscle T2 values of patients with IIM were compared to those of volunteers. The average T2 value of patients with IIM was 47.4 ± 9.0 ms, much higher than that of healthy controls (33.9 ± 0.9 ms) with a *p*-value of less than 0.001. Figure 2 presents the ROC curve for the diagnostic performance of T2 mapping in the differentiation of IIM from healthy controls. The area under the curve (AUC) was 0.986 (*p* < 0.001) with a cutoff value of 36.4 ms (sensitivity 96.9%, and specificity 100%).

### Muscle T2 map segmented using deep learning well-delineated active muscular inflammation in IIM

**Fig. 3:**
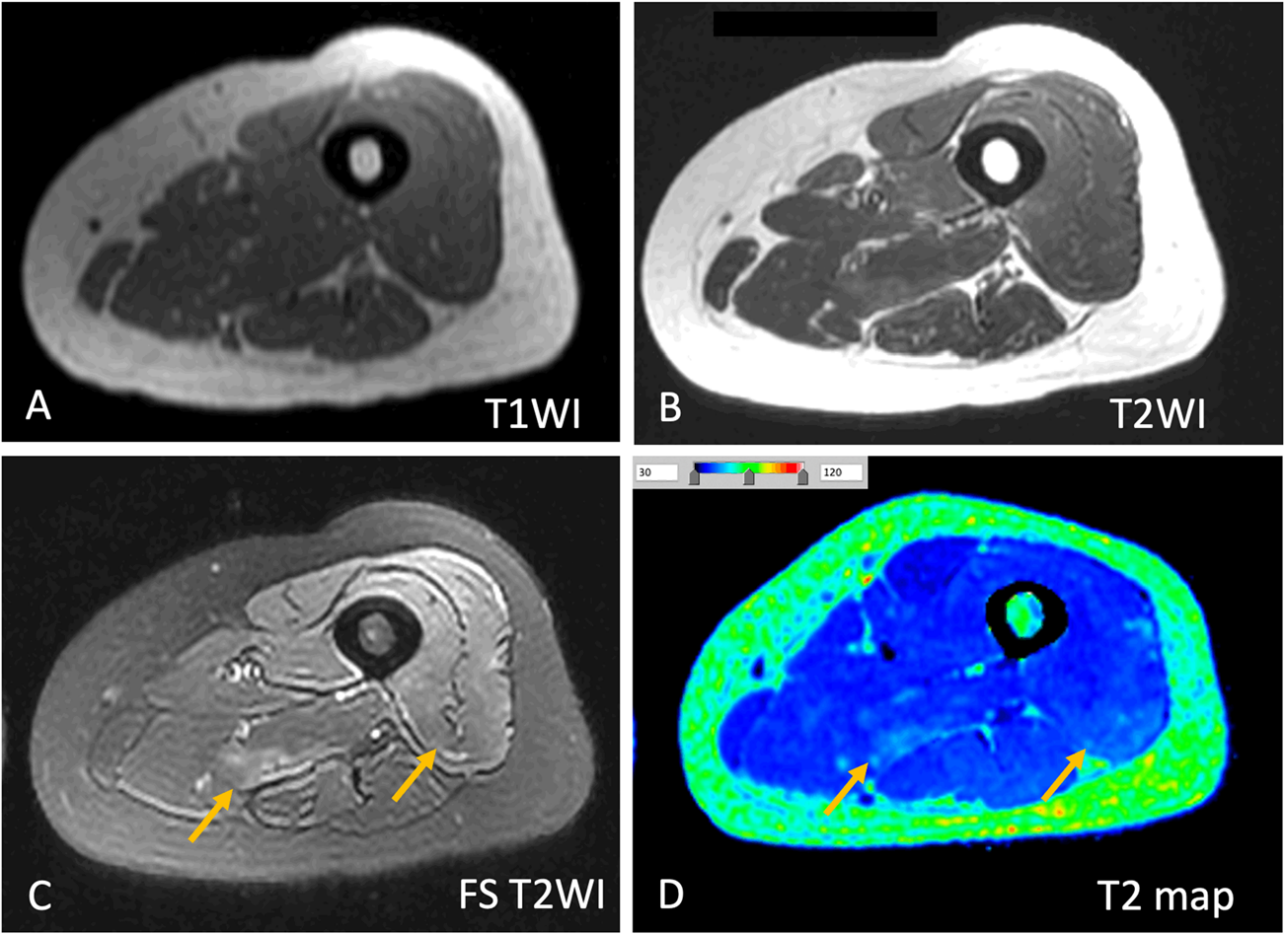
MRI of a 52-year-old female with dermatomyositis. (**A**) Axial T1-weighted image (T1WI), (**B**) T2WI, (**C**) fat-saturated (FS) T2WI, and (**D**) color-coded T2 map. The serum muscle enzymes of this patient were normal, while slight hyperintensity of quadriceps and adductor magnus (arrows) was noticed on FS T2WI which was further confirmed by an elevation of T2 value (44.9 ms) presenting as light blue in color-coded T2 map

To further depict muscle T2 values in patients with IIM, this quantitative imaging parameter was compared to serum muscle enzymes (Table 1). Results showed that T2 value, an indicator of active muscle inflammation [6, 7], positively correlated with all the serum muscle enzymes, including CK, ALT, AST, and LDH (*p* = 0.003, 0.016, 0.006, and 0.015, respectively). Of note, if we use 36.4 ms as the cutoff value, T2 map detected muscle disease even in 21 of 22 (95.5%, 21/22) patients with normal serum CK levels, six of whom underwent muscle biopsy after MRI and were confirmed to have muscle inflammation histologically. Taking Fig. 3 as an example, a patient first thought to have amyopathic DM with normal muscle enzymes and no evidence of muscle involvement or symptoms was diagnosed with hypomyopathic DM (no objective weakness but evidence of subclinical muscle involvement) after MRI. In this patient, abnormal skeletal muscle signal intensities were found on fat-saturated T2WI, which was confirmed by mild elevations in the T2 value (41.0 ms).

**Table 1:** Correlation of T2 values with clinical and laboratory parameters in patients with idiopathic inflammatory myopathy

Metric	Normal range	No.	Mean (range)	*r*_s_	*p*
Age	—	64	44.3 years (19–72 years)	0.091	0.476
Disease duration	—	64	22.2 months (1–144 months)	0.242	0.054
CK	24–195 U/L	64	1961 U/L (15–17156 U/L)	0.376	0.003**
ALT	9–50 U/L	64	90 U/L (11–536 U/L)	0.306	0.016*
AST	15–40 U/L	64	110 U/L (15–742 U/L)	0.345	0.006**
LDH	0–250 U/L	64	500 U/L (143–1675 U/L)	0.307	0.015*

*CK* creatine kinase, *ALT* alanine aminotransferase, *AST* aspartate aminotransferase, *LDH* lactate dehydrogenase. * represents *p* < 0.05, and ** represents *p* < 0.01

From HRCT results, an ILD score (range: 0–8), the sum of severity and extent of ILD based on the Likert scoring system, was used to semi-quantify IIM-associated ILD. Thirty-one patients (48%, 31/64) were found to have ILD, while no honeycombing was observed. The average ILD score was 4.8 ± 1.6. There was no association between ILD scores and muscle T2 values (*r*_s_ = 0.226, *p* = 0.080), and the T2 values of patients without ILD and those with ILD were similar (49.3 ± 10.5 ms vs 45.3 ± 6.5 ms, *p* = 0.118). Eight patients with ASS and an average ILD score of 4.75 were diagnosed; this was markedly higher than 2.11 seen in those without ASS (*Z* = 2.556, *p* = 0.011). In addition, patients were sub-grouped based on whether ASS was present; there was no difference of muscle T2 values in the subgroup analysis (*Z* = 0.771, *p* = 0.440).

## Discussion

Muscle weakness, the most typical characteristic of IIM, develops in a subacute way. Thus, T2 mapping, with its ability to detect early symptoms and quantify muscle inflammation, is useful in the diagnosis and monitoring of the disease. Compared to manual measurements, muscle T2 measurement based on DL segmentation is more accurate, efficient, and cost-effective, because all measurements can be taken simultaneously and automatically, and the error rate is much lower [15]. Our results showed that T2 values segmented by DL automatically in patients with IIM were markedly higher than those in volunteers, with a cutoff value of 36.4 ms (sensitivity 96.9%, and specificity 100%). In addition, T2 values segmented by DL in patients with IIM were positively correlated with all the serum muscle enzymes, and T2 mapping might be able to detect muscle disease in patients with normal muscle enzyme levels.

Using combined incremental and federated learning approaches, we could improve an existing segmentation algorithm without transmitting data from protected patients to a third party. Recently, federated learning approaches were proposed for the automated segmentation of brain [16] and lung [17] lesions. In our case, the automated segmentation was implemented inside a graphical user interface that allowed instant manual validation and refinement and continuous learning with every dataset [18]. OpenSAFELY, a new data analytics platform created by Goldacre et al, was developed to address epidemiology and treatment of COVID-19 in England [19]. It involves a detailed real-time analysis of primary care patient records within that data center and keeping a log of all queries that the group ran on the records. This concept was designed and built to promote both research and patient confidentiality at the same time. We used a similar approach in our study. Artificial intelligence (AI) requires large quantities of well-curated data to learn about diseases with sufficient accuracy; the safety of data and patients presents a challenge. However, by using federated learning [20], our MRI bilateral thigh muscle data were stored and analyzed by DL in situ in our medical center, while we only pooled the trained algorithm parameters — not the data — to the central server. That is to say, data sharing was limited to an improvement in the algorithm, which was sent to the developer in another country and analyzed by researchers worldwide. This new methodology makes it possible to get results without transmitting primary data and without sharing large, sensitive datasets.

The pathologic manifestations of DM include perivascular atrophy, a hallmark of this disease, and other abnormalities, including complement deposition on endomysial capillaries and decreased capillary density. However, the cellular infiltrates predominate within fascicles, and inflammatory cells invade individual muscle fibers in PM cases [21]. The above changes in necrosis, degeneration, regeneration, and inflammation of muscles result in increased amounts of intracellular and extracellular free water, which is quantified using T2 mapping, a measurement of T2 relaxation times [22]. Our results indicate that T2 mapping automatically segmented by DL can differentiate diseased muscles of IIM from normal muscles of healthy controls. Previous studies found increased T2 values in edematous muscles of patients with IIM showed increased signal intensities on FS T2WI images compared with those of unaffected muscles, which were isointense [7]. However, the apparently unaffected muscles of patients with IIM also exhibited elevated T2 values, confirmed by the findings of mild inflammation in biopsies [8]. Thus, these findings should be taken into account when performing T2 measurements. In contrast to small manually placed ROIs, T2 mapping segmented by DL can cover all thigh muscles simultaneously, which should increase the clinical utility of this technique.

The increased serum muscle enzymes were indispensable criteria in both the 1975 Bohan and Peter criteria and 2017 EULAR/ACR guidelines [23], where CK was found to be the most sensitive and most commonly tested and followed analyte. We found a significant correlation between muscle T2 values segmented by DL and serum muscle enzymes including CK levels in patients with IIM, which is in line with previous findings. In clinical practice, CK levels can be normal in about 20% of patients with DM even in those patients with weakness [24, 25]. Persistently low serum muscle enzyme levels when obvious muscle weakness is present could also occur in patients with advanced disease and significant loss of muscle mass. Therefore, in these patient groups, assessments other than muscle enzyme levels can be beneficial, including muscle MRI, typically performed on the bilateral thigh muscles with T2 mapping [26, 27]. In the present study, patients with normal serum CK levels were found to have increased T2 values. In other words, T2 values generated by T2 mapping, with the advent of DL, might be used as an alternative marker to assess active IIM muscle inflammation.

The first major contributor to morbidity of patients with IIM is muscular involvement, which is followed by IIM-associated ILD. The prevalence of ILD in patients with IIM varies widely (from 39 to 74%), depending on the case series and methods used to identify ILD [28, 29]. As such, about 48% (31/64) of patients were found to have ILD based on HRCT in our cohort. Although IIM-associated ILD is indistinguishable from the idiopathic ILD and ILD that occurs in other systemic rheumatic diseases on histopathology, honeycomb formations are less frequently observed [30], which is consistent with our finding that no honeycomb formations were seen in this study. Moreover, the dissociation between the severity of muscular involvement and ILD which was observed in clinical practice is first proven quantitatively herein in our study, reflecting the complicated and vaguely understood pathogenesis of IIM. Therefore, both muscular and pulmonary involvement should be assessed for the diagnosis and follow-up during treatment of this disease entity [31]. T2 mapping automatically segmented by DL provides a measurable noninvasive marker that shows changes in the degree of inflammation. In other words, decreases in muscle T2 values might be considered treatment targets.

There were several limitations to our study. First, besides inflammation, fatty infiltration in advanced IIM also contributes to increased muscle T2 values. Previous researchers have tried to correct for these fat infiltrates on T2 maps using a biexponential model but did not find the theoretical advantage of fat-corrected T2 values [26]. In the present study, the impact of fat was minimized by applying chemical-shift-selective fat saturation during T2 map data acquisition [32]. Second, T2 values segmented by DL were not compared with manual ROI or segmentation measurements because manual ROI placement only covers a small area of thigh muscles, which could not represent the entire thigh muscle. Additionally, manual muscle segmentation would take hours for one patient and is not practical. In addition, the DL algorithm only performs segmentation and defines the masks, which would not change the T2 values. Last but not least, the volunteers’ age and female-to-male ratio were not matched with patients, but we believe this was a minor confounder.

In conclusion, T2 mapping of thigh muscle using automatic DL segmentation could differentiate diseased muscles of IIM from normal muscles of healthy controls, and shows promise in the assessment of active muscular inflammation of IIM.

## Supplementary information

ESM 1 (DOCX 306 kb)

## Abbreviations

ALT: Alanine aminotransferase
ASS: Antisynthetase syndrome
AST: Aspartate aminotransferase
CK: Creatine kinase
DL: Deep learning
DM: Dermatomyositis
HRCT: High-resolution computed tomography
IIM: Idiopathic inflammatory myopathy
ILD: Interstitial lung disease
LDH: Lactate dehydrogenase
MRI: Magnetic resonance imaging
MSA: Myositis-specific autoantibodies
PM: Polymyositis
ROI: Regions of interest

## Declarations

### Guarantor

The scientific guarantor of this publication is Prof. Zhengyu Jin.

### Statistics and biometry

No complex statistical methods were necessary for this paper.

### Methodology

• retrospective

• cross-sectional study

• performed at one institution
