# Supplementary material for: Assessment of idiopathic inflammatory myopathy using a deep learning method for muscle T2 mapping segmentation
Source: Eur Radiol. 2022 Nov 18;33(4):2350–7. doi: 10.1007/s00330-022-09254-9 (PMC9672653; doi:10.1007/s00330-022-09254-9)
Supplement: Supplementary file 1 — (DOCX 306 kb) [file 330_2022_9254_MOESM1_ESM.docx]

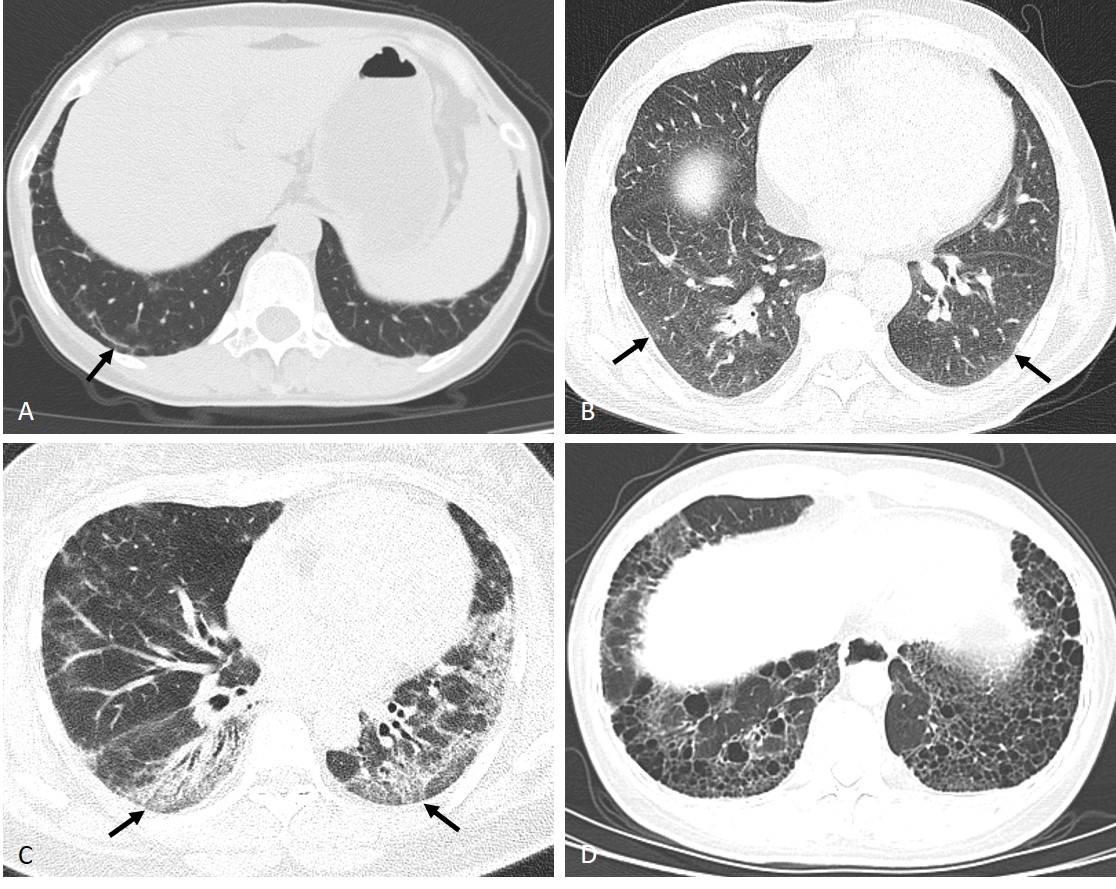


**Supplementary Fig. 1** The 5-point Likert score to grade the severity of interstitial lung disease (ILD) with score 0 representing no ILD. (A) Score 1, minimal thickening of interlobular septa forming subpleural bands (arrow); (B) Score 2, mild reticulation and ground glass opacity (arrows); (C) Score 3, moderate reticulation and ground glass opacity (arrows); and (D) Score 4, honeycombing.
